# Supplementary material for: The Impact of YRNAs on HNSCC and HPV Infection
Source: Biomedicines. 2023 Feb 23;11(3):681. doi: 10.3390/biomedicines11030681 (PMC10045647; doi:10.3390/biomedicines11030681)
Supplement: Supplementary file 1 [file biomedicines-11-00681-s001.zip › biomedicines-2127464-supplementary.pdf]

Supplementary Table S1. Different clinical and pathological properties (T stage, N stage, clinical stage, clusters and localization) of whole group of patients' from GEO database in terms of YRNA1, YRNA3, YRNA4 and YRNA5 expressions.

		YRNA1		YRNA3		YRNA4		YRNA5	
		**Mean ± SEM**	***P* value**	**Mean ± SEM**	***P* value**	**Mean ± SEM**	***P* value**	**Mean ± SEM**	***P* value**
**T stage**	T1, n=35	7.699 ± 0.07563	0.6708	8.145 ± 0.04585	**0.0045**	7.024 ± 0.07172	0.8238	6.727 ± 0.04421	0.0699
	T2, n=80	7.734 ± 0.05268		8.179 ± 0.02991		6.996 ± 0.04650		6.728 ± 0.02241	
	T3, n=58	7.818 ± 0.06685		8.317 ± 0.03348		6.979 ± 0.04664		6.651 ± 0.02206	
	T4, n=97	7.787 ± 0.04874		8.241 ± 0.02571		7.025 ± 0.04176		6.701 ± 0.01814	
	T1+T2, n=115	7.723 ± 0.04312	0.2291	8.169 ± 0.02498	**0.0019**	7.005 ± 0.03887	0.531	6.728 ± 0.02048	0.1238
	T3+T4, n=155	7.799 ± 0.03933		8.270 ± 0.02054		7.008 ± 0.03138		6.682 ± 0.01413	
**N stage**	N0, n=94	7.681 ± 0.04882	**0.0426**	8.179 ± 0.02986	0.0865	7.018 ± 0.04028	0.4306	6.697 ± 0.01875	0.4081
	N1, n=32	7.733 ± 0.08296		8.289 ± 0.04754		6.903 ± 0.05759		6.713 ± 0.04166	
	N2, n=132	7.841 ± 0.04059		8.251 ± 0.02157		7.026 ± 0.03643		6.706 ± 0.01694	
	N3, n=12	7.710 ± 0.1751		8.167 ± 0.05368		6.975 ± 0.1369		6.651 ± 0.06750	
	N0+N1, n=126	7.694 ± 0.04196	**0.011**	8.207 ± 0.02561	0.2527	6.989 ± 0.03361	0.5776	6.701 ± 0.01746	0.9086
	N2+N3, n=144	7.830 ± 0.03986		8.244 ± 0.02032		7.022 ± 0.03515		6.702 ± 0.01649	
**Clinical Stage**	I, n=18	7.788 ± 0.1199	0.4849	8.115 ± 0.07607	**0.0020**	7.155 ± 0.1181	0.4483	6.710 ± 0.03825	0.2652
	II, n=37	7.706 ± 0.07700		8.107 ± 0.04377		7.052 ± 0.06945		6.732 ± 0.03188	
	III, n=37	7.683 ± 0.07506		8.299 ± 0.04248		6.922 ± 0.05309		6.687 ± 0.03883	
	IV, n=177	7.792 ± 0.03639		8.248 ± 0.01894		7.000 ± 0.03003		6.697 ± 0.01448	
	I+II, n=55	7.733 ± 0.06455	0.4885	8.110 ± 0.03816	**0.0002**	7.086 ± 0.06035	0.2329	6.725 ± 0.02467	0.0982
	III+IV, n=215	7.775 ± 0.03271		8.257 ± 0.01723		6.986 ± 0.02640		6.695 ± 0.01364	
**Clusters**	Classical, n=30	7.435 ± 0.06887	**< 0.0001**	8.251 ± 0.04359	**0.0006**	6.806 ± 0.04903	**0.0051**	6.649 ± 0.03202	**0.0318**
	Basal, n=84	7.647 ± 0.04906		8.186 ± 0.02922		6.998 ± 0.04440		6.671 ± 0.02041	
	Atypical, n=73	8.010 ± 0.05514		8.328 ± 0.03250		7.035 ± 0.04997		6.718 ± 0.02406	
	Mesenchymal, n=83	7.793 ± 0.04832		8.170 ± 0.02595		7.063 ± 0.04340		6.736 ± 0.02198	
**Localization**	Oral cavity, n=83	7.733 ± 0.04528	0.1123	8.238 ± 0.02653	0.2699	6.987 ± 0.03806	0.7024	6.690 ± 0.01904	0.4513
	Hypopharynx, n=33	7.870 ± 0.09710		8.238 ± 0.05683		7.062 ± 0.07307		6.740 ± 0.03524	
	Larynx, n=48	7.650 ± 0.06966		8.156 ± 0.04066		7.021 ± 0.06394		6.676 ± 0.02734	
	Oropharynx, n=102	7.811 ± 0.04951		8.240 ± 0.02465		6.994 ± 0.04217		6.708 ± 0.02156	

Supplementary Table S2. Expression analysis of YRNA1, YRNA3, YRNA4 and YRNA5 among GEO database patients in terms of age, smoking status, HPV status, HPV type, viral status and p53 mutation status. Differential criteria, numbers of cases and p values are included in the table.

			**YRNA1**	**YRNA3**	**YRNA4**	**YRNA5**
**Age**	ALL	<=60, n = 153	0.2747	0.3164	0.8098	0.5427
		>60, n = 117				
	HPV(+)	<=60, n = 40	0.7104	0.7187	0.3433	0.1853
		>60, n = 33				
	HPV(-)	<=60, n = 112	0.2892	0.3814	0.3863	0.7418
		>60, n = 84				
**Smoking**	ALL	No/Ex, n = 48	0.3218	0.2661	0.8107	0.1707
		Yes, n = 222				
	HPV(+)	No/Ex, n = 21	0.5303	0.2533	0.2597	0.7467
		Yes, n = 52				
	HPV(-)	No/Ex, n = 27	0.5345	0.5763	0.1729	0.1263
		Yes, n = 169				
**HPV**	ALL	Positive, n = 73	0.4921	0.8671	0.9846	0.6368
		Negative, n = 195				
**HPV Type**	ALL	p16, n = 60	0.9483	0.7363	0.4754	0.8008
		Other, n = 13				
**Viral status**	ALL	DNA+RNA+, n = 35	0.5261	0.4007	1.000	0.1922
		DNA+RNA-, n = 19				
**p53 Mutation Status**	ALL	WT, n = 142	0.440	0.6940	0.0529	0.4679
		Disruptive, n = 51				
		Non-disruptive, n = 33				
	HPV(+)	WT, n = 47	0.9245	0.7088	0.9526	0.5547
		Disruptive, n = 8				
		Non-disruptive, n = 4				
	HPV(-)	WT, n = 95	0.0177	0.5469	0.0669	0.5946
		Disruptive, n = 43				
		Non-disruptive, n = 29				

Supplementary Table S3. Different clinical and pathological properties (T stage, N stage, clinical stage, clusters and localization) of HPV(-) group of patients' from GEO database in terms of YRNA1, YRNA3, YRNA4 and YRNA5 expressions.

		YRNA1		YRNA3		YRNA4		YRNA5	
		**Mean ± SEM**	***P* value**	**Mean ± SEM**	***P* value**	**Mean ± SEM**	***P* value**	**Mean ± SEM**	***P* value**
**T stage**	T1, n=25	7.736 ± 0.09818	0.6712	8.127 ± 0.05721	**0.0110**	7.078 ± 0.09229	0.8592	6.700 ± 0.02901	**0.0437**
	T2, n=53	7.681 ± 0.06108		8.141 ± 0.03810		7.009 ± 0.05429		6.742 ± 0.02972	
	T3, n=46	7.814 ± 0.07691		8.321 ± 0.04001		6.965 ± 0.05127		6.642 ± 0.02587	
	T4, n=72	7.771 ± 0.05949		8.257 ± 0.03019		7.008 ± 0.04495		6.698 ± 0.02176	
	T1+T2, n=78	7.699 ± 0.05180	0.3077	8.136 ± 0.03152	**0.0003**	7.031 ± 0.04708	0.851	6.729 ± 0.02223	**0.0430**
	T3+T4, n=118	7.788 ± 0.04692		8.282 ± 0.02420		6.991 ± 0.03385		6.676 ± 0.01680	
**N stage**	N0, n=81	7.670 ± 0.05342	0.0996	8.177 ± 0.03257	0.1884	7.028 ± 0.04400	0.4765	6.698 ± 0.02059	0.836
	N1, n=24	7.729 ± 0.09705		8.250 ± 0.05641		6.909 ± 0.07319		6.695 ± 0.03343	
	N2, n=83	7.835 ± 0.05179		8.267 ± 0.02916		7.011 ± 0.04045		6.699 ± 0.02119	
	N3, n=8	7.805 ± 0.2594		8.177 ± 0.06878		7.043 ± 0.2025		6.684 ± 0.1011	
	N0+N1, n=105	7.683 ± 0.04664	**0.0193**	8.193 ± 0.02827	0.0975	7.001 ± 0.03799	0.603	6.697 ± 0.01755	0.7851
	N2+N3, n=91	7.832 ± 0.05187		8.259 ± 0.02731		7.014 ± 0.04051		6.698 ± 0.02105	
**Clinical Stage**	I, n=16	7.853 ± 0.1253	0.4807	8.149 ± 0.08117	**0.0204**	7.218 ± 0.1239	0.2016	6.741 ± 0.03528	0.1995
	II, n=31	7.680 ± 0.08512		8.100 ± 0.05054		7.067 ± 0.07573		6.731 ± 0.03651	
	III, n=28	7.677 ± 0.08709		8.273 ± 0.05049		6.936 ± 0.06708		6.668 ± 0.03376	
	IV, n=121	7.775 ± 0.04552		8.254 ± 0.02408		6.980 ± 0.03293		6.690 ± 0.01760	
	I+II, n=47	7.739 ± 0.07075	0.7929	8.117 ± 0.04292	**0.0022**	7.118 ± 0.06543	0.0795	6.735 ± 0.02668	**0.047**
	III+IV, n=149	7.757 ± 0.04043		8.258 ± 0.02167		6.972 ± 0.02950		6.686 ± 0.01560	
**Clusters**	Classical, n=27	7.429 ± 0.07567	**< 0.0001**	8.250 ± 0.04677	0.2605	6.811 ± 0.05398	**0.0224**	6.646 ± 0.03522	**0.01**
	Basal, n=70	7.651 ± 0.05649		8.193 ± 0.03121		6.992 ± 0.05005		6.679 ± 0.02274	
	Atypical, n=38	7.958 ± 0.07841		8.276 ± 0.04809		7.060 ± 0.06876		6.744 ± 0.03671	
	Mesenchymal, n=61	7.785 ± 0.05349		8.182 ± 0.03151		7.051 ± 0.05142		6.759 ± 0.02696	
**Localization**	Oral cavity, n=70	7.752 ± 0.04995	0.1737	8.251 ± 0.02879	0.4842	6.999 ± 0.04160	0.5243	6.695 ± 0.02105	0.2719
	Hypopharynx, n=30	7.886 ± 0.1056		8.218 ± 0.05998		7.084 ± 0.07844		6.753 ± 0.03779	
	Larynx, n=45	7.636 ± 0.07088		8.169 ± 0.04194		7.006 ± 0.06261		6.672 ± 0.02719	
	Oropharynx, n=47	7.769 ± 0.07975		8.227 ± 0.04185		6.963 ± 0.05832		6.683 ± 0.03009	

Supplementary Table S4. Different clinical and pathological properties (T stage, N stage, clinical stage, clusters and localization) of HPV(+) group of patients' from GEO database in terms of YRNA1, YRNA3, YRNA4 and YRNA5 expressions.

		YRNA1		YRNA3		YRNA4		YRNA5	
		**Mean ± SEM**	***P* value**	**Mean ± SEM**	***P* value**	**Mean ± SEM**	***P* value**	**Mean ± SEM**	***P* value**
**T stage**	T1, n=10	7.606 ± 0.1002	0.597	8.190 ± 0.07527	0.5312	6.890 ± 0.09192	0.5335	6.794 ± 0.1398	0.8168
	T2, n=27	7.836 ± 0.09863		8.254 ± 0.04503		6.971 ± 0.08862		6.699 ± 0.03172	
	T3, n=12	7.835 ± 0.1383		8.301 ± 0.05426		7.033 ± 0.1137		6.683 ± 0.03974	
	T4, n=24	7.822 ± 0.08387		8.195 ± 0.05084		7.081 ± 0.1024		6.704 ± 0.03381	
	T1+T2, n=37	7.774 ± 0.07806	0.5115	8.237 ± 0.03839	0.9019	6.949 ± 0.06889	0.1499	6.725 ± 0.04359	0.675
	T3+T4, n=36	7.826 ± 0.07131		8.230 ± 0.03889		7.065 ± 0.07721		6.697 ± 0.02587	
**N stage**	N0, n=13	7.751 ± 0.1204	0.5334	8.191 ± 0.07673	0.1265	6.954 ± 0.1005	0.9403	6.690 ± 0.04574	0.3018
	N1, n=8	7.746 ± 0.1704		8.409 ± 0.07701		6.883 ± 0.07665		6.768 ± 0.1384	
	N2, n=48	7.845 ± 0.06710		8.223 ± 0.03123		7.055 ± 0.07208		6.718 ± 0.02894	
	N3, n=4	7.520 ± 0.07293		8.147 ± 0.09712		6.840 ± 0.07620		6.584 ± 0.01296	
	N0+N1, n=21	7.749 ± 0.09623	0.5464	8.274 ± 0.05949	0.3455	6.927 ± 0.06778	0.6303	6.720 ± 0.05834	0.8693
	N2+N3, n=52	7.820 ± 0.06325		8.217 ± 0.02967		7.038 ± 0.06716		6.707 ± 0.02716	
**Clinical Stage**	I, n=2	7.267 ± 0.08565	0.2166	7.846 ± 0.1133	**0.0449**	6.657 ± 0.1089	0.4457	6.454 ± 0.03065	0.0646
	II, n=6	7.841 ± 0.1852		8.144 ± 0.07465		6.972 ± 0.1866		6.739 ± 0.06174	
	III, n=37	7.702 ± 0.1566		8.378 ± 0.07478		6.879 ± 0.06772		6.747 ± 0.1238	
	IV, n=177	7.830 ± 0.05989		8.234 ± 0.02981		7.043 ± 0.06295		6.711 ± 0.02561	
	I+II, n=8	7.697 ± 0.1658	0.4217	8.070 ± 0.07635	**0.0330**	6.894 ± 0.1474	0.3016	6.668 ± 0.06522	0.7573
	III+IV, n=65	7.812 ± 0.05578		8.254 ± 0.02817		7.020 ± 0.05535		6.716 ± 0.02745	
**Clusters**	Classical, n=3	7.490 ± 0.1271	**0.0075**	8.261 ± 0.1399	**0.0020**	6.762 ± 0.08059	0.2714	6.678 ± 0.05534	0.9492
	Basal, n=13	7.639 ± 0.09184		8.158 ± 0.08818		7.008 ± 0.1007		6.652 ± 0.04636	
	Atypical, n=35	8.068 ± 0.07733		8.385 ± 0.04186		7.008 ± 0.07351		6.689 ± 0.03030	
	Mesenchymal, n=22	7.815 ± 0.1081		8.135 ± 0.04441		7.095 ± 0.08209		6.674 ± 0.03323	
**Localization**	Oral cavity, n=13	7.630 ± 0.1057	0.4221	8.169 ± 0.06772	**0.0434**	6.919 ± 0.09584	0.9115	6.661 ± 0.04485	0.6299
	Hypopharynx, n=3	7.715 ± 0.1749		8.444 ± 0.1532		6.838 ± 0.1412		6.607 ± 0.04403	
	Larynx, n=3	7.858 ± 0.3790		7.962 ± 0.1460		7.241 ± 0.4668		6.725 ± 0.1893	
	Oropharynx, n=54	7.842 ± 0.06279		8.252 ± 0.02931		7.024 ± 0.06158		6.728 ± 0.03110	

Supplementary Table S5. GSEA results of YRNA3, YRNA4 and YRNA5 in HPV(+) and HPV(-) groups. The table shows enriched pathways in which YRNAs were found and normalized enrichment score (NES), nominal *p* value (NOM p-val) and false discovery rate q value (FDR q-val); *p* value < 0.05 and FDR q value < 0.25 considered as significant.

**YRNA3 HPV-**			
**NAME**	**NES**	**NOM *p*-val**	**FDR q-val**
GOBP NEGATIVE REGULATION OF TELOMERE MAINTENANCE VIA TELOMERE LENGTHENING	-1.9782459	0.0	0.053096812
HP RECURRENT SINOPULMONARY INFECTIONS	-1.8658305	0.0	0.12829725
GOMF MODIFICATION DEPENDENT PROTEIN BINDING	-1.8820041	0.0	0.12906608
HP OVERWEIGHT	-1.8980998	0.0	0.14073196
GOBP REGULATION OF DNA TEMPLATED TRANSCRIPTION ELONGATION	-1.8234807	0.0	0.15425658
GOBP NEGATIVE REGULATION OF TELOMERE MAINTENANCE	-1.8324279	0.0019047619	0.1582201
HP CHRONIC RHINITIS	-1.7905881	0.0020325202	0.1612035
GOBP EXTRACELLULAR TRANSPORT	-1.8120512	0.0	0.16179463
HP RHINITIS	-1.8412032	0.001992032	0.16405647
GOMF METHYLATED HISTONE BINDING	-1.7934369	0.0	0.17126898
GOMF TAU PROTEIN KINASE ACTIVITY	-1.7998508	0.0	0.17340714
GOBP REGULATION OF MRNA PROCESSING	-1.7601594	0.012145749	0.17780732
HP TYPE I DIABETES MELLITUS	-1.7604265	0.0	0.18889232
GOBP REGULATION OF TRANSCRIPTION ELONGATION FROM RNA POLYMERASE II PROMOTER	-1.76461	0.002008032	0.18889286
HP SACCADIC SMOOTH PURSUIT	-1.7723002	0.004016064	0.196775
GOMF LYSINE ACETYLATED HISTONE BINDING	-1.766147	0.004115226	0.19932006
GOCC PML BODY	-1.7444333	0.0	0.20205322
GOBP MITOTIC G2 M TRANSITION CHECKPOINT	-1.7472899	0.0	0.20507146
GOBP REGULATION OF MRNA 3 END PROCESSING	-1.7233238	0.01183432	0.2371359
HP RECURRENT MYCOBACTERIAL INFECTIONS	-1.7241713	0.00967118	0.24631071
**YRNA3 HPV+**			
GOMF ELECTRON TRANSFER ACTIVITY	1.8677815	0.0	0.19593209
GOMF OXIDOREDUCTASE ACTIVITY ACTING ON NAD P H QUINONE OR SIMILAR COMPOUND AS ACCEPTOR	1.764012	0.0019267823	0.19837508
GOCC MITOCHONDRIAL PROTEIN CONTAINING COMPLEX	1.7686732	0.002008032	0.20060508
GOBP ATP SYNTHESIS COUPLED ELECTRON TRANSPORT	1.7705008	0.004032258	0.2146511
GOMF OXIDOREDUCTASE ACTIVITY ACTING ON NAD P H	1.7719706	0.0019607844	0.23302357

GOBP RESPIRATORY ELECTRON TRANSPORT CHAIN	1.7767512	0.002004008	0.23910148
GOCC INNER MITOCHONDRIAL MEMBRANE PROTEIN COMPLEX	1.7828401	0.0020283975	0.24707568
HALLMARK OXIDATIVE PHOSPHORYLATION	1.7626885	0.002004008	0.005301828
HALLMARK REACTIVE OXYGEN SPECIES PATHWAY	1.4093591	0.03968254	0.24114163
EIF4E UP	1.649572	0.0076045627	0.08992033
JAK2 DN.V1 DN	-1.701562	0.0	0.021776728
**YRNA4 HPV+**			
HP MUSCLE ABNORMALITY RELATED TO MITOCHONDRIAL DYSFUNCTION	-1.7900214	0.003816794	0.08160625
GOCC MITOCHONDRIAL PROTEIN CONTAINING COMPLEX	-1.7933891	0.001984127	0.084746495
GOCC RIBOSOME	-1.7576778	0.0020120724	0.086412735
GOCC INNER MITOCHONDRIAL MEMBRANE PROTEIN COMPLEX	-1.7612879	0.0019880715	0.086973555
GOCC SM LIKE PROTEIN FAMILY COMPLEX	-1.7641017	0.005905512	0.08813139
HP ABNORMAL ACTIVITY OF MITOCHONDRIAL RESPIRATORY CHAIN	-1.7795303	0.001984127	0.08942255
HP DECREASED ACTIVITY OF MITOCHONDRIAL COMPLEX IV	-1.7957678	0.0	0.090028845
GOBP PSEUDOURIDINE SYNTHESIS	-1.7517582	0.002016129	0.09041234
HP DECREASED ACTIVITY OF MITOCHONDRIAL COMPLEX I	-1.7663393	0.003868472	0.09097942
GOCC ORGANELLAR RIBOSOME	-1.7440696	0.003984064	0.092969164
HP FOCAL T2 HYPERINTENSE BASAL GANGLIA LESION	-1.7405037	0.0019646366	0.094048716
GOMF RNA POLYMERASE ACTIVITY	-1.7681264	0.0020120724	0.094050676
GOCC MITOCHONDRIAL LARGE RIBOSOMAL SUBUNIT	-1.7460752	0.004008016	0.09429248
GOCC SPLICEOSOMAL TRI SNRNP COMPLEX	-1.7720566	0.0	0.0952907
GOBP RESPIRATORY ELECTRON TRANSPORT CHAIN	-1.7224374	0.006	0.09538635
HP ABNORMAL MITOCHONDRIA IN MUSCLE TISSUE	-1.7243658	0.0019493178	0.09586323
HP CYTOCHROME C OXIDASE NEGATIVE MUSCLE FIBERS	-1.7192985	0.009746589	0.09651434
GOCC RESPIRASOME	-1.7246213	0.001980198	0.09916524
GOMF STRUCTURAL CONSTITUENT OF RIBOSOME	-1.7967312	0.0020449897	0.099606596
GOCC RNA POLYMERASE III COMPLEX	-1.7262846	0.004016064	0.10052045
HP INCREASED CSF LACTATE	-1.8023745	0.0	0.10266626
HP PROXIMAL TUBULOPATHY	-1.7271383	0.001984127	0.10312337
GOMF OXIDOREDUCTION DRIVEN ACTIVE TRANSMEMBRANE TRANSPORTER ACTIVITY	-1.710967	0.004	0.10659187
GOBP ATP SYNTHESIS COUPLED ELECTRON TRANSPORT	-1.7274623	0.003968254	0.10700925
GOBP RRNA MODIFICATION	-1.7291517	0.015968064	0.10883787

GOBP MATURATION OF LSU RRNA	-1.703576	0.008316008	0.11107196
HP ABNORMAL BRAINSTEM MRI SIGNAL INTENSITY	-1.7047994	0.007920792	0.11288348
GOCC SMALL RIBOSOMAL SUBUNIT	-1.6922555	0.004048583	0.11615897
HP FOCAL T2 HYPERINTENSE BRAINSTEM LESION	-1.6871963	0.013257576	0.118535146
GOCC LARGE RIBOSOMAL SUBUNIT	-1.8028729	0.0	0.11889576
GOBP RIBONUCLEOPROTEIN COMPLEX SUBUNIT ORGANIZATION	-1.6963247	0.00203666	0.11906513
GOMF NADH DEHYDROGENASE ACTIVITY	-1.6884307	0.009689922	0.11932154
GOCC NADH DEHYDROGENASE COMPLEX	-1.6922985	0.007858546	0.119328335
HP ABNORMAL CSF METABOLITE LEVEL	-1.6932342	0.0019455253	0.120735504
GOBP RIBOSOMAL LARGE SUBUNIT ASSEMBLY	-1.6787145	0.014256619	0.123646736
GOBP MITOCHONDRIAL ELECTRON TRANSPORT NADH TO UBIQUINONE	-1.6763805	0.007984032	0.12521635
HP MITOCHONDRIAL MYOPATHY	-1.6743815	0.007797271	0.1259581
GOBP OXIDATIVE PHOSPHORYLATION	-1.6791629	0.001980198	0.12612931
HP DECREASED ACTIVITY OF THE PYRUVATE DEHYDROGENASE COMPLEX	-1.680187	0.01369863	0.12710579
GOCC SPLICEOSOMAL SNRNP COMPLEX	-1.8080812	0.001992032	0.12964629
GOBP RNA 5 END PROCESSING	-1.6680386	0.018518519	0.1349184
HP ABNORMAL BASAL GANGLIA MRI SIGNAL INTENSITY	-1.8183807	0.0	0.13517973
GOBP RIBOSOMAL LARGE SUBUNIT BIOGENESIS	-1.8318129	0.0	0.14006335
GOBP RIBOSOME ASSEMBLY	-1.6560718	0.014830508	0.15311626
HP PROGRESSIVE SPASTIC PARAPLEGIA	-1.6572673	0.007858546	0.1538089
GOBP NADH DEHYDROGENASE COMPLEX ASSEMBLY	-1.6537895	0.01734104	0.15487425
GOCC SNO S RNA CONTAINING RIBONUCLEOPROTEIN COMPLEX	-1.642935	0.020325202	0.16865261
GOBP MITOCHONDRIAL RESPIRATORY CHAIN COMPLEX ASSEMBLY	-1.6440282	0.019267824	0.16942422
GOCC ORGANELLE INNER MEMBRANE	-1.6445376	0.004	0.17163943
GOBP SPLICEOSOMAL SNRNP ASSEMBLY	-1.8730546	0.0020325202	0.20264482
HP HEMANGIOBLASTOMA	-1.6278477	0.005882353	0.20333087
GOCC RIBOSOMAL SUBUNIT	-1.8321669	0.0	0.20796385
GOCC CYTOCHROME COMPLEX	-1.6158822	0.0121951215	0.23196703
GOBP RIBONUCLEOPROTEIN COMPLEX BIOGENESIS	-1.6103685	0.022403259	0.23990421
GOCC RESPIRATORY CHAIN COMPLEX IV	-1.6084716	0.009861933	0.2415121
GOBP MATURATION OF 5 8S RRNA FROM TRICISTRONIC RRNA TRANSCRIPT SSU RRNA 5 8S RRNA LSU RRNA	-1.6110382	0.022357723	0.24231188
GOBP MATURATION OF 5 8S RRNA	-1.6047502	0.012658228	0.2439163
HP ABNORMALITY OF THE INTERVERTEBRAL DISK	-1.606121	0.0077669905	0.24414927
HALLMARK DNA REPAIR	-1.6215535	0.004166667	0.05216014
**YRNA5 HPV+**			

HALLMARK TGF BETA SIGNALING	1.6527486	0.003960396	0.07703276
HALLMARK HEDGEHOG SIGNALING	1.5475082	0.025948104	0.1820822
HALLMARK APICAL JUNCTION	1.3922218	0.054435484	0.22485684
HALLMARK UV RESPONSE DN	1.4895577	0.031746034	0.24095283
HALLMARK WNT BETA CATENIN SIGNALING	1.3985876	0.083333336	0.24317823

Supplementary Table S6. List of genes derived from NGS analysis of modified FaDu and Detroit562 cell lines with YRNA1 overexpressing plasmid found in infectious disease pathways.

Genes from FaDu	Genes from FaDu	Genes from Detroit562	Genes from Detroit562
ACTB	*RPL35*	*HLA-B*	*GNAS*
HMGA1	*RPL41*	*HLA-C*	*RPS29*
NPM1	*RPL7A*	*CAV1*	*YWHAE*
RPL10	*RPLP1*	*RPS3A*	*RPS15A*
RPL24	*RPS16*	*RPS2*	*RPS27A*
RPL30	*RPS20*	*HLA-A*	*RPL29*
RPL39	*RPS6*	*RPS6*	*PPIA*
RPL6	*TUBA1B*	*UBC*	*RPL35*
RPLP0	*ENO1*	*RPS14*	*ENO1*
RPS14	*HSP90AB1*	*RPS16*	*RPL32*
RPS2	*RAN*	*B2M*	*RPL10*
RPS3A	*RPL18A*	*RPS10*	*CALR*
SLC25A5	*RPL3*	*RPS13*	*TUBA1B*
XRCC6	*RPL38*	*RPL26*	*RPLP1*
ACTG1	*RPL5*	*RPL41*	*RPLP0*
HSP90AA1	*RPL8*	*RPS9*	*RPL4*
PPIA	*RPS11*	*NPM1*	*HLA-H*
RPL12	*RPS18*	*HSP90AB1*	*RPL8*
RPL29	*RPS25*	*ACTB*	*HSP90AA1*
RPS8	*UBC*		

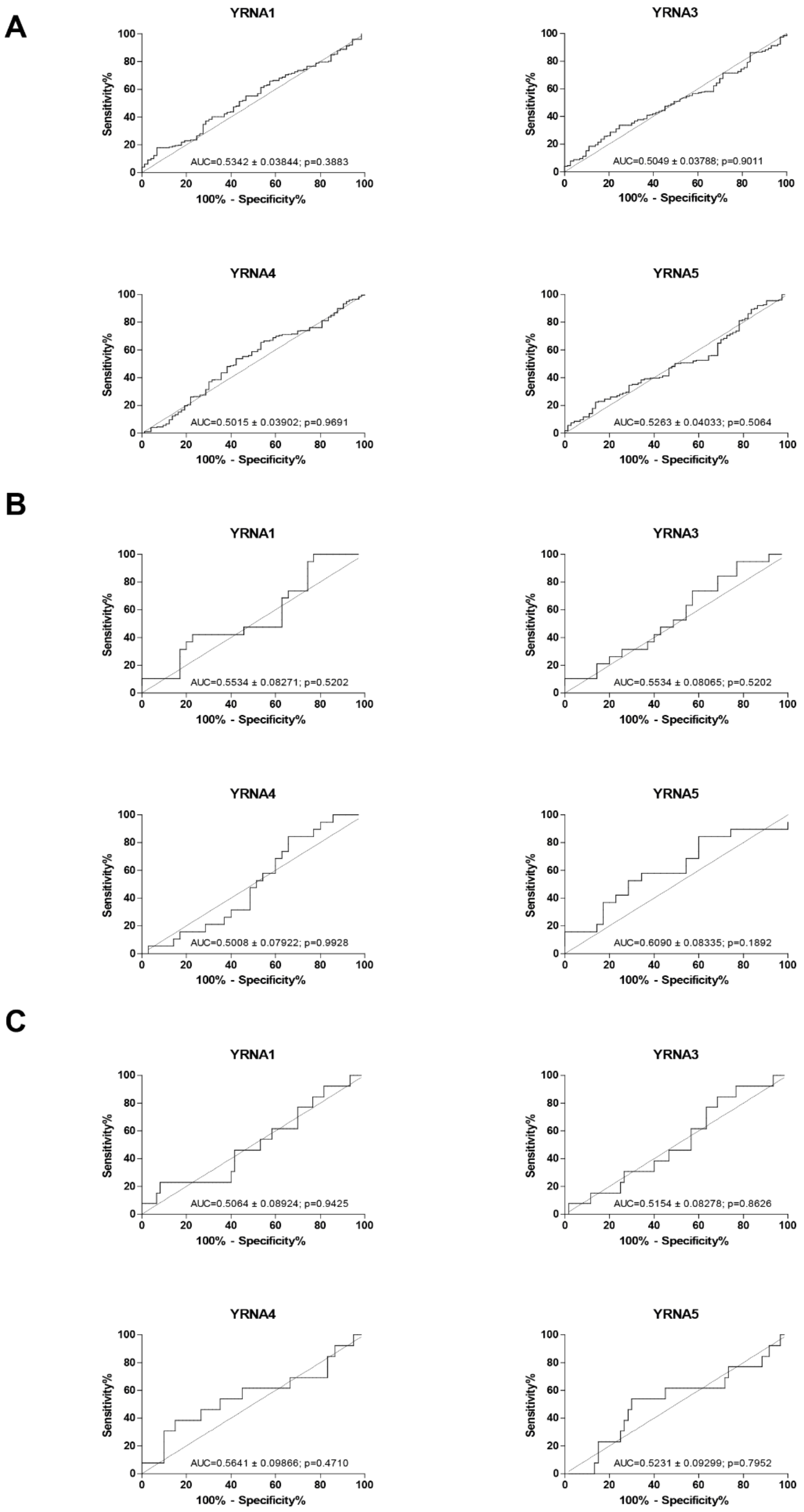

Supplementary Figure S1. ROC Analysis of different parameters among YRNAs: A) HPV(+) vs HPV(-); B) DNA+RNA+ vs DNA+RNA-; C) p16 vs other HPV.

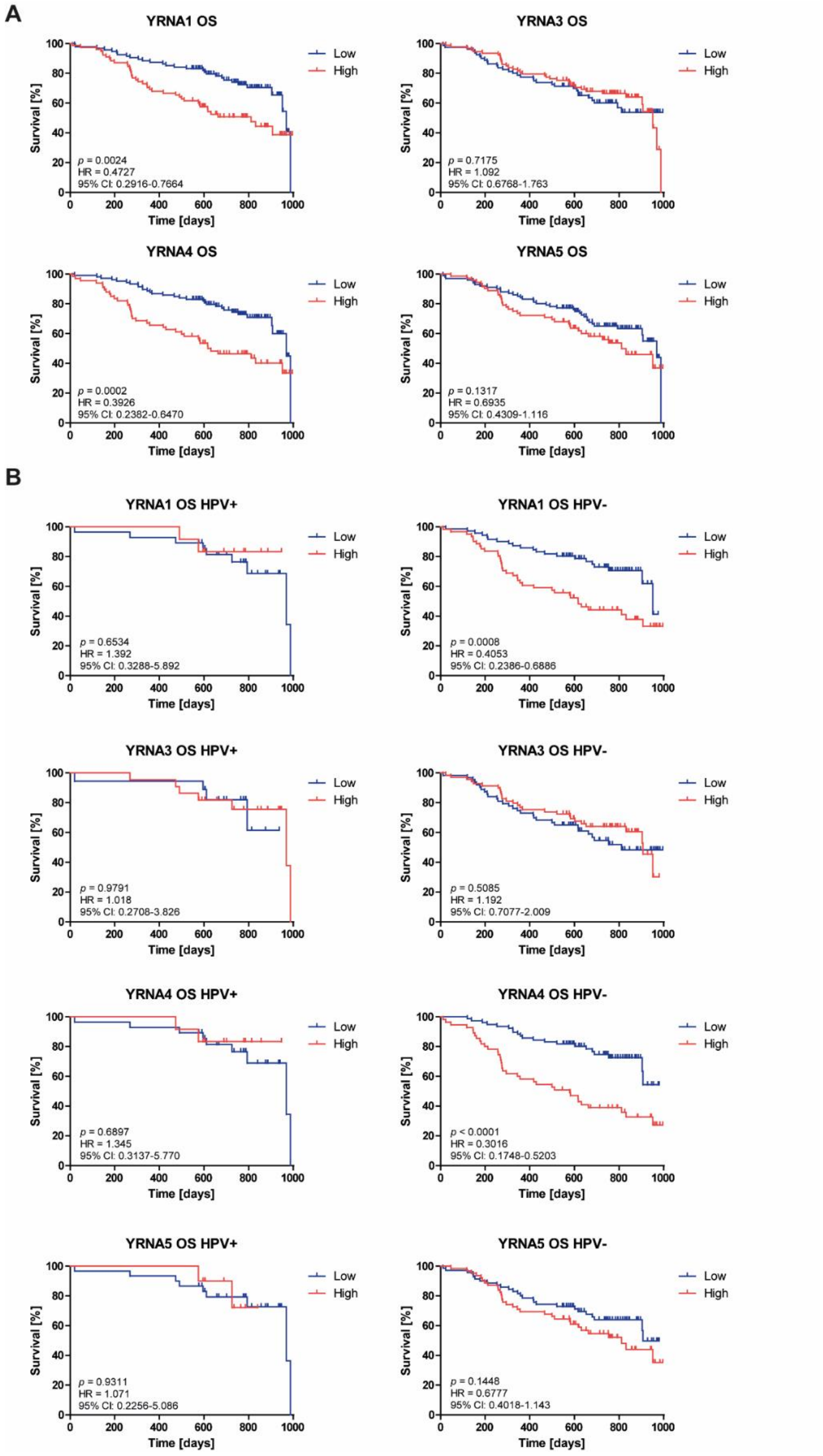

Supplementary Figure S2. Association between expression level of YRNA1, YRNA3, YRNA4, and YRNA5, and HNSCC patients' overall survival in 1000 days time in: (A) whole group of patients (HPV(-) and HPV(+)), (B) in only HPV(+) and in only HPV(-). Low and high expression groups of patients were divided based on mean as a cut-off calculated separately for every of the groups, **all patients** (YRNA1: low n = 152, high n = 117; YRNA3: low n = 128, high n = 142; YRNA4: low n = 174, high n = 96; YRNA5 low n = 164, high n =

106), **HPV(+)** (YRNA1: low n = 48, high n = 25; YRNA3: low n = 36, high n = 37; YRNA4: low n = 50, high n = 23; YRNA5 low n = 50, high n = 23), **HPV(-)** (YRNA1: low n = 112, high n = 84; YRNA3: low n = 94, high n = 102; YRNA4: low n = 123, high n = 73; YRNA5 low n = 111, high n = 85).

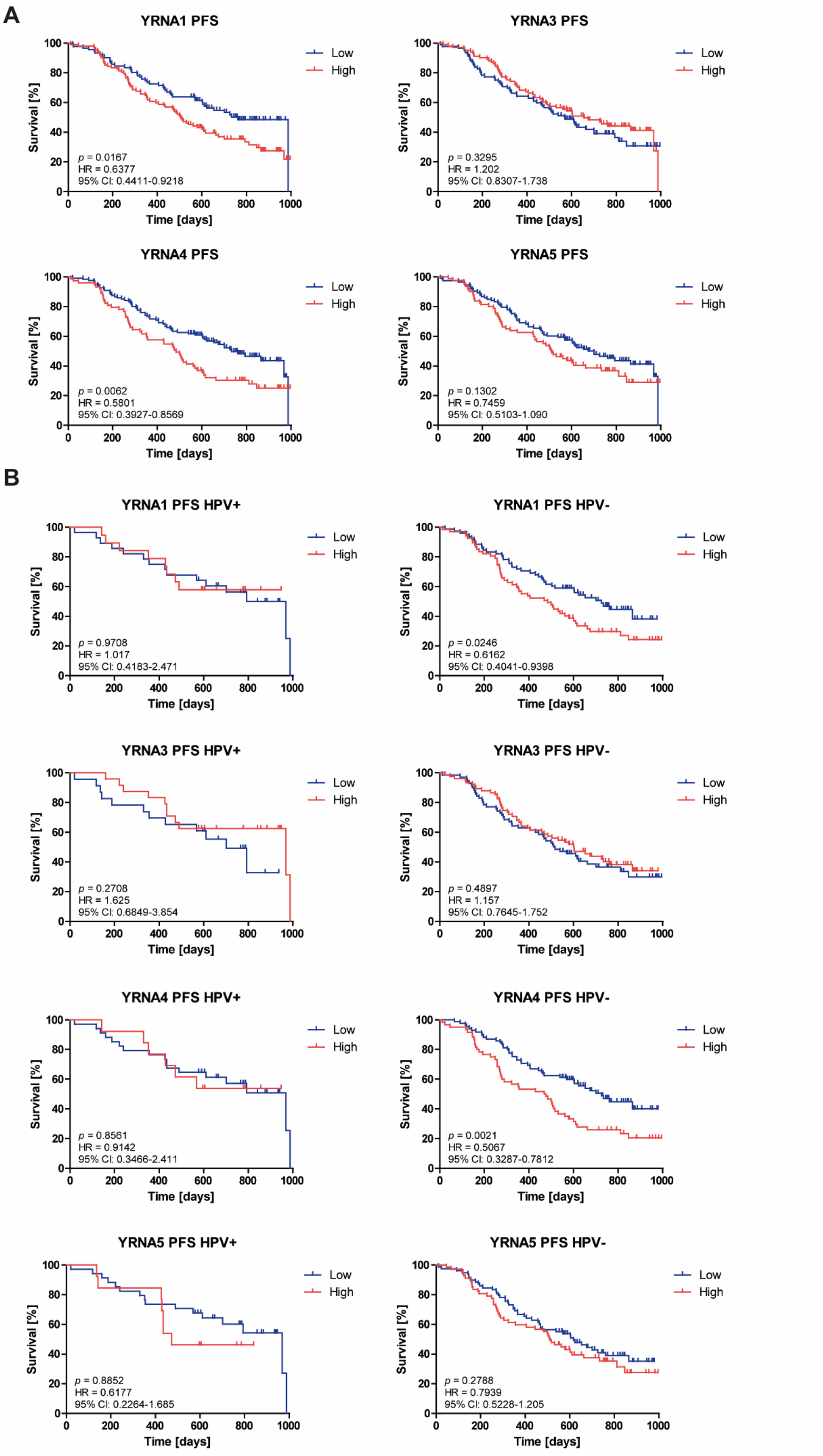

Supplementary Figure S3. Association between expression level of YRNA1, YRNA3, YRNA4, and YRNA5, and HNSCC patients' progression-free survival in 1000 days time in:

(A) whole group of patients (HPV(-) and HPV(+)), (B) in only HPV(+) and in only HPV(-). Low and high expression groups of patients were divided based on mean as a cut-off calculated separately for every of the groups: **all patients** (YRNA1: low n = 132, high n = 138; YRNA3: low n = 128, high n = 142; YRNA4: low n = 174, high n = 96; YRNA5 low n = 164, high n = 106), **HPV(+)** (YRNA1: low n = 39, high n = 34; YRNA3: low n = 36, high n = 37; YRNA4: low n = 50, high n = 23; YRNA5 low n = 50, high n = 23), **HPV(-)** (YRNA1: low n = 112, high n = 84; YRNA3: low n = 94, high n = 102; YRNA4: low n = 123, high n = 73; YRNA5 low n = 110, high n = 86).

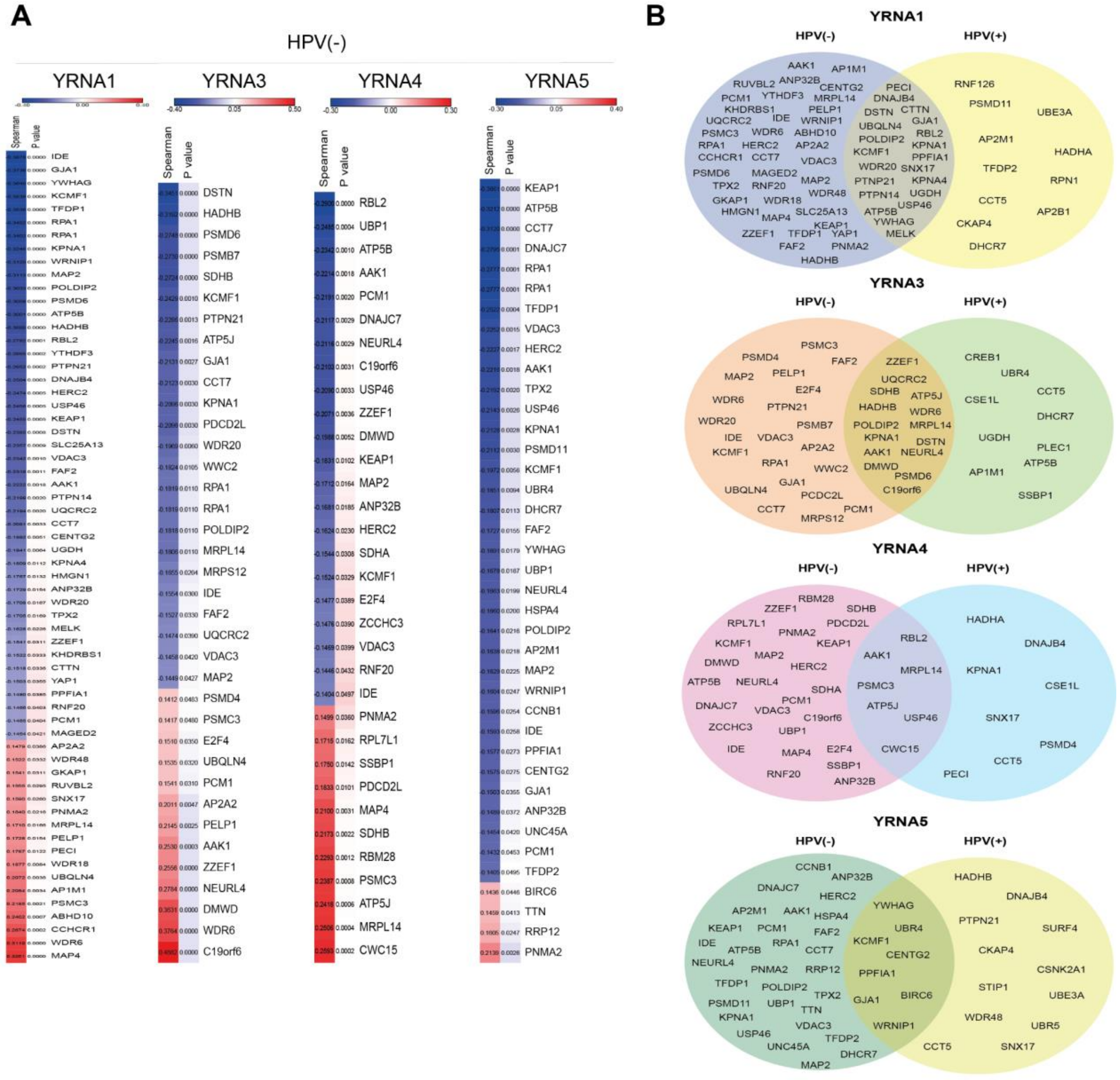

Supplementary Figure S4. Analysis of genes correlated with YRNAs based on GEO database. A) Heat maps of genes correlated with YRNAs in HPV(-) group; B) Venn Diagrams depicting gene distribution expression between HPV(+) and HPV(-) groups among YRNAs.
